# Crisis checklists for in-hospital emergencies: expert consensus, simulation testing and recommendations for a template determined by a multi-institutional and multi-disciplinary learning collaborative

**DOI:** 10.1186/s12913-017-2288-y

**Published:** 2017-05-08

**Authors:** Christian P. Subbe, John Kellett, Paul Barach, Catriona Chaloner, Hayley Cleaver, Tim Cooksley, Erik Korsten, Eilish Croke, Elinor Davis, Ashley JR De Bie, Lesley Durham, Chris Hancock, Jilian Hartin, Tracy Savijn, John Welch, on behalf of the Crisis Checklist Collaborative, Paul Barach, Helene Beaugrand, Dorothy Breen, Declan Byrne, Catriona Chalmers, Hayley Cleaver, Tim Cooksley, Eilish Croke, Elinor Davis, Ashley De Bie, Peter Donnelly, Eímhín Dunne, Lesley Durham, Bryn Ellis, Rohan Goel, Chris Hancock, Jillian Hartin, Denise Hinge, Mark Holland, Dirk Hueske-Kraus, John Kellett, Sean Kennelly, Erik Korsten, Geoffrey Lighthall, Rebecca Lunn, Michael Müller, Clodagh O’Dwyer, Kerian O’Mahony, Nigel Paice, Lisa Roberts, Tracy Savijn, Christian P. Subbe, Dafydd Thomas, Richard Walsh, Frank Weber, John Welch, Simon Woodworth

**Affiliations:** 1 Ysbyty Gwynedd & Bangor University, Penrhosgarnedd, Bangor, LL57 2PW UK; 2 Nenagh Hospital, Nenagh, Ireland; 3 Wayne State University School of Medicine, Detroit, MI USA; 4 Ysbyty Glan Clwyd, Rhyl, UK; 5 Cardiff University School of Medicine, Cardiff, UK; 6 The Christie Hospital, Manchester, UK; 7 Catharina Ziekenhuis, Eindhoven, The Netherlands; 8 Acute Medicine Program, Dublin, Ireland; 9 North of England Critical Care Network (NoECCN), North Tyneside General Hospital, North Shields, UK; 10 Public Health Wales, Cardiff, UK; 11 University College London Hospitals, London, UK

**Keywords:** Rapid response teams, Crisis, Reliability, Patient safety, Simulation, Learning Collaborative

## Abstract

**Background:**

‘Failure to rescue’ of hospitalized patients with deteriorating physiology on general wards is caused by a complex array of organisational, technical and cultural failures including a lack of standardized team and individual expected responses and actions. The aim of this study using a learning collaborative method was to develop consensus recomendations on the utility and effectiveness of checklists as training and operational tools to assist in improving the skills of general ward staff on the effective rescue of patients with abnormal physiology.

**Methods:**

A scoping study of the literature was followed by a multi-institutional and multi-disciplinary international learning collaborative. We sought to achieve a consensus on procedures and clinical simulation technology to determine the requirements, develop and test a safe using a checklist template that is rapidly accessible to assist in emergency management of common events for general ward use.

**Results:**

Safety considerations about deteriorating patients were agreed upon and summarized. A consensus was achieved among an international group of experts on currently available checklist formats performing poorly in simulation testing as first responders in general ward clinical crises. The Crisis Checklist Collaborative ratified a consensus template for a general ward checklist that provides a list of issues for first responders to address (i.e. ‘Check In’), a list of prompts regarding common omissions (i.e. ‘Stop & Think’), and, a list of items required for the safe “handover” of patients that remain on the general ward (i.e. ‘Check Out’). Simulation usability assessment of the template demonstrated feasibility for clinical management of deteriorating patients.

**Conclusions:**

Emergency checklists custom-designed for general ward patients have the potential to guide the treatment speed and reliability of responses for emergency management of patients with abnormal physiology while minimizing the risk of adverse events. Interventional trials are needed.

## Background

Failure to detect and treat clinical deterioration, either from a medical condition or due to a complication of surgical treatment is a common life threatening problem [1]. Hospitals have introduced Rapid Response Systems (RRS), which use an increasingly standardized evaluation and escalation treatment paradigm to manage patients with physiological derangements [2, 3]. In contrast, the efferent limb clinical response is much more variable and ranges from the patient’s primary care team, to lone nurse practitioners, to dedicated Rapid Response Teams (RRTs) with intensive care, medical, nursing and allied care providers. The first responders of the efferent limb on general medical and surgical wards will nearly always be an ad hoc assembly of available providers with limited experience in managing common emergency situations .

The publication of ‘To Err is Human’ in 2000 [4] has prompted a systems-approach towards safe care including applying human factors tools from safety-critical industries, such as aviation and nuclear power, that can be used to mitigate propagation of process failure to systems failures and adverse patient events [5]. Checklists have been used effectively as part of routine safety procedures [6]. The introduction of the Safer Surgery Checklist [7] required operating theatre teams in 2009 to change their behaviour in team readiness and has been credited with the reduction of post-operative complications and mortality [8]. Studies describing the use of checklists beyond the highly controlled environments of the Intensive Care Unit (ICU) and the operating theatre are rare: the SURPASS trial in the Netherlands demonstrated a slight reduction in mortality associated with the use of multiple checklists during the surgical patient pathway [9]. Implementation of a ‘sepsis six’ care bundle in general ward areas using a checklist format has also demonstrated only a small reduction in mortality [10]. However, Urbach et al., found that surgical checklists had little impact when clinicians were not involved in checklist design or implementation [11].

Aviation distinguishes between ‘normal’, ‘non-normal’ and ‘emergency’ checklists [6]. ‘Normal’ checklists are used as part of standard operating procedures. They include lists used for preparation of a flight or technical checks by maintenance staff. The World Health Organisation’s surgical checklist can be seen as a ‘normal’ checklist. Similar checklists have been used to effectively implement central venous catheter insertion [12] and ventilator associated pneumonia prevention ‘care bundles’ in many ICUs. [13] In these highly controlled settings, checklists seem to have reduced mortality and adverse events and helped to sustain improvements once embedded in clinical practice [8, 12]. ‘Emergency’ checklists deal with uncommon, and unexpected crisis situations likely to have catastrophic outcomes. There are guidelines for the format and content of ‘emergency’ checklists that specify the recommended colours and typefaces to use [14]. In intensive care and surgery the checklists are intended to be used by several people working together in close partnership [15].

While Medical Emergency Team call-out criteria and Early Warning Scores have helped to standardize the recognition of deterioration it is not clear how response could be standardized. We aimed to provide clinicians with rapidly accessible standardized checklists to assist structuring standardized responses to patient deterioration using a checklist format. These exoplored how checklists could be designed to be used by the patient’s ‘home’ teams and help to structure emergency management and team response to common emergencies during escalation to Rapid Response teams.

## Methods

### Aim

The aim of this study was to develop consensus recommendations on the development and safe testing of a checklist template designed to manage common emergencies that occur on general medical wards by a multi-institutional and multi-disciplinary learning collaborative.

### Design: the crisis checklists learning collaborative

A learning collaborative is an innovative and comprehensive approach to multidisciplinary ‘action research’ that unite researchers, clinicians and policy makers to create a “community of practice” [16]. The Crisis Checklists Learning Collaborative came together to create a safe, learning environment for advancing knowledge and promoting best practices related to developing and implementing better care for the deteriorating patient. The group consisted of 32 multidisciplinary experts with over 200 years of combined clinical experience, currently involved in research and clinical practice related to emergency checklists, were invited to participate in a series of consensus meetings. All participants were based at tertiary care medical centers and universities. The invitees attended three face-to-face meetings in Ireland (Dublin April 3-4th, 2014), Wales (Bangor September 5-6th 2014) and England (Manchester January 30-31st 2015).

Our cooperative learning facilitated the accomplishment of a specific end product using the principles of co-design with clinicians and research scientists working together with clinician end-users and patient representatives. Of the 38 participants, 16 were senior medical professionals, nine nursing professionals, six had a technical background while six were in training. Participants included nurse and medical practitioners in the area of Rapid Response Systems [4], Intensive Care [4], Anesthesiology [2], acute medical [4] and general ward care (6,) as well as a patient representative. The skill set included national and regional program managers for Rapid Response Systems and Acute Care [4], human factors and patient safety specialists, including those with military and aviation experience, and experts in information technology, quality improvement, systems and graphic design [6] and medical students [2]. Members of the group were from France [1], Germany [3], Ireland [9], Netherlands [2], United Kingdom [17] and the United States of America [2].

### Literature search strategy

A scoping study of the literature [3] on checklists and their current use in medical care was performed by three members of the group (JK, CS, PB), prior to the face-to-face meeting, with the aim of summarizing existing research findings and identifying key gaps in the existing literature. We searched for published articles in medical and non-medical literature that assessed the effects of that assessed the effects of checklists. The studies were reviewed for their research design and internal validity. We assessed each study’s findings in regard to their effects on patient mortality, morbidity, patient safety, as well as process outcomes. We searched MEDLINE, EMBASE, CRD, for all studies on use of safety checklists. Reference lists of selected articles were searched for potentially relevant studies meeting the inclusion criteria (snowballing). In addition, we used Google search engine using the search words checklist, rapid response team, resuscitation and patient safety. Protocols and publications that outlined safety criteria for use of checklists for deteriorating patients on medical wards were identified and distributed to the group. Additionally, any publication or protocol that a member of the crisis checklist learning collaborative deemed important was circulated prior to the meeting.

### Ethics approval

Advice from the Health Research Authority (HRA) was sought with regard to the classification of the study. The HRA classified the collaborative as ‘Not Research’. The waiver for informed consent was confirmed by the Bangor Research and Development office.

### Workflow learning events

At the first meeting additional knowledge was contributed by participants via presentations from individual group members of any published or unpublished checklist data; further discussions, debate and critique were exchanged in a series of facilitated workshops and focus groups until clear agreement was reached. At the end of the first meeting the following tasks were assigned to designated conference participants:

1. Determine the common clinical situations on general wards for which checklists might be suitable by a further review of the literature;
2. Survey experts and active practitioners in rapid response strategies and systems; and
3. Draft prototype checklists for candidate conditions based on the templates of the Operating Room (OR) Crisis Checklists at www.projectcheck.org/crisis (courtesy to adriadnelab, https://www.ariadnelabs.org/).

Following the face-to-face meeting, a summary of the safety criteria for checklists was drafted, and, using an iterative process, was circulated to panel members via email until the group had reached consensus or agreed that they could not reach consensus. Consensus was defined as 100% agreement amongst the group.

The second meeting pilot-tested the checklists for validity and reliability in a high fidelity clinical Simulation Suite at the Ysbyty Gwynedd Hospital, in Bangor, Wales [16, 18, 19]. Test clinical scenarios were undertaken as part of learning events and were administered to teams of volunteer candidates (i.e., doctors, nurses, medical and nursing students) access to medical notes, observation and medication charts and a ‘nurse’ facilitator delivered the information about their simulated ‘patient’. Clinical scenarios were run twice in a randomized fashion, with and without the use of checklists. The performance of teams and individual candidates, with and without, the use checklists was observed, analysed and constructively criticized by the expert participants.

The third collaborative meeting provided the feedback and debate on checklist design, usage, and assessed the role that the clinical culture played in both medical and non-medical settings. Different checklists designs were discussed, piloted, reworked, amended and modified through multiple iterations via discussions, debate and critique in a series of facilitated workshops and focus groups. A consensus on the clinical issues to be addressed by checklists on general medical wards, and the design of the template for these checklists, was ratified by the conference participants. The checklists were edited by a graphic designer and pilot tested with physicians, students and nurses in the Simulation Suite of the Ysbyty Gwynedd Hospital, Bangor, Wales during several sessions in May, and June 2015. Participants self-assessed teamwork, task management, decision making and communication using Likert scales with and without checklists.

## Results

The literature search found only two references relating to the use of emergency checklists in operating rooms [18, 20], and, we found no references related to the use of checklists and care bundles for emergencies outside intensive care units and operating theatres. We found no published reports evaluating emergency checklist usage on general hospital wards, and no checklists designed for this purpose.

### Selection of rapid response team scenarios suitable for checklists

We reviewed published data on the acuity of general ward patients that Rapid Response teams were commonly called on to evaluate. An analysis of 400 calls to a RRT in an Australian Hospital demonstrated that six patient scenario types were responsible for the bulk of call-outs: hypoxia (41%), hypotension (28%), altered conscious state (23%), tachycardia (19%), increased respiratory rate (14%) and oliguria (8%) [19]. Clinicians responding to a deteriorating patient could therefore potentially be directed to a limited catalogue of checklists to act upon when treating a deteriorating patient.

A semi-structured survey was designed, piloted, refined, and given to faculty and international specialists in the field from Europe, the US and Australia, to identify candidate conditions for checklists at the International Society for Rapid Response Systems (iSRRS) in Miami in May 2014. A catalogue of candidate conditions amenable to checklists was generated from the survey responses (Table 1).

**Table 1:** Candidate simulation conditions

Group	Example conditions
Checklists based on Operating Room Crisis Checklists [18]	• Anaphylaxis • Airway • Advanced Life Support scenarios
Interventional crisis	• Gastrointestinal bleed • Myocardial Infarction • Sepsis • Acute Kidney Injury • Fast Atrial Fibrillation
Diagnostic crisis	• Respiratory distress • Un-specifically unwell • Altered mental status
Objective signs of instability	National Early Warning Score (NEWS [17]) level 3, NEWS level 5, NEWS level 7

The resulting catalogue of candidate checklists was tested for face validity at the Ysbyty Gwynedd Hospital, a 500 bed facility in the UK. Patients that fulfilled national trigger criteria for a rising National Early Warning Score (NEWS) [17] of 6 or more were reviewed on three general medical wards over a 4 week period. We found 32 patients had new abnormalities, while 68% could be meaningfully allocated to three of the 11 pre-defined scenarios of ‘respiratory distress’ (38%), ‘sepsis’ (15%) and ‘Altered Loss of Consciousness’ (15%).

### Simulation testing of emergency checklists templates (Bangor workshop)

The consensus view on currently available checklist formats is that for most providers the use of checklists might bring a ‘task-based’ rather than a “thought-based” approach to patient management and might result in a failure to seek and consider all available information. For example, in the ‘Respiratory Distress’ scenario the expected diagnosis of pulmonary embolism was not considered by several candidates. General ward checklists, therefore, need to be designed to prompt comprehensive data gathering and provoke appropriate thought as well as action. Checklist formats similar to the operating theatre checklists [18], require a team of several responders already at the bedside. However, in a general ward the first-responder is often a lone responder, most likely a registered nurse and/or a junior doctor with limited experience in managing emergencies. The group’s consensus view was that emergency checklists for general wards needed to be modified and be consistent with the organizational structure, cultural context and available resources at the time the RRT is called [21].

### Ratification of a checklist template based on consensus opinion (Manchester meeting)

**Fig. 1:**
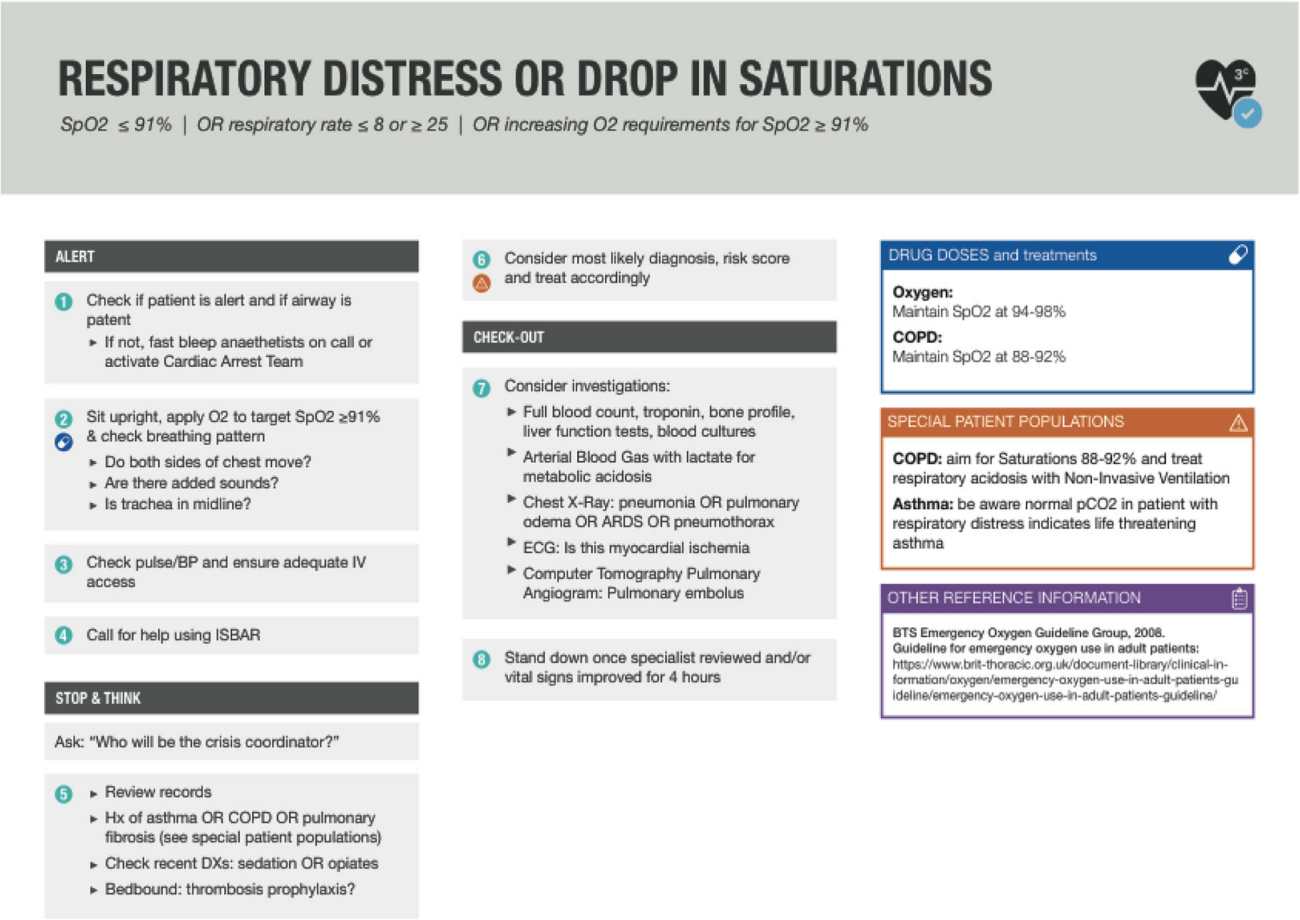
Sample Checklist. Template courtesy to adriadnelab (https://www.ariadnelabs.org/). Based on the OR Crisis Checklists at http://www.projectcheck.org/crisis. All reasonable precautions have been taken to verify the information contained in this publication. The responsibility for the interpretation and use of the materials lies with the reader

We came to a consensus at the third Collaborative meeting that a general ward checklist should provide a list of key issues for the first responders of the patient’s team (home team) to address (labelled the ‘*Check In’*), a list of prompts for further actions or appropriate escalation (labelled ‘*Stop & Think’*), and how they might structure the Rapid Response team intervention, and then list the items required for the safe “hand-off” of patients who had been stabilised and remained on the general ward (labelled ‘*Check Out’*). We retained the checklist item addressing team leadership (‘Who will be the crisis coordinator’) from the crisis checklists for the operating room. Our expectation is that this role is either taken up by the most senior clinician or delegated by the same. Twelve candidate checklists were written applying these principles and a graphic designer edited the final version of the checklists for clarity and usability (Fig. 1).

### Simulation testing of the ratified consensus checklist template

The suite of checklists that addressed the candidate medical emergencies (Table 1) was tested by volunteer candidates (i.e. doctors, nurses, medical and nursing students) in simulated environments at five hospitals (Bangor, Cork, Manchester, Rhyl and Eindhoven) in a standardized manner. Several volunteer responders reported usability problems with the checklists due to lack of familiarity. A short video clip that summarized the rationale and principles of Emergency Checklist was developed [22] to aid training and facility with the simulation mechanics.

When clinical teams were asked to assess their performance during patient management of common simulated emergencies they felt that the use of checklists improved their team work (*p* < 0.016) and communication (*p* < 0.01) and overall performance (*p* < 0.034).

## Discussion

The aim of this study was to develop consensus recommendations to provide clinicians about the safe use of emergency checklists to assist in the emergency management of deteriorating patients on general medical wards. Hospitalized patient deterioration continues to challenge healthcare providers with variable outcomes and ongoing preventable harm(ref?). Utilizing previous evidence, simulation testing and expert opinion, the learning collaborative group achieved consensus on the best templates to use for RRT teams to assist in structuring patient management when faced with treating deteriorating patients on general medical wards.

This project used an established learning collaborative methodology to gain consensus on developing custom designed and rapidly accessible checklists for ward patients using standard procedures and clinical simulation technology to improve patient management. We found that general wards are qualitatively different from other clinical areas because the first responders must use resources that are available and therefore cannot rely on guidance by specialists. Our experience using a simulated environment suggests that while traditional checklist templates are not appropriate for general ward use, an innovative and flexible template we developed may be of value for the management of the common deteriorating patient by producing rapidly accessible and more reliable responses with improved measures of teamwork.

The systematic assessment of patient physiology at the bedside has led to dramatic reductions in rates of cardiopulmonary arrests [23–25]. Despite this success many instances of abnormal patient physiology do not lead to early activation of a RRT [26–29]. Moreover, even when a RRT team is called key interventions may be missed [29], possibly as a result of errors in mental modelling and/or an incomplete understanding on how to respond to patterns of abnormal patient physiology [30]. Consequently a significant proportion of patients that trigger a RRT response subsequently generate recurrent “call-outs” [31]. A potential solution for these challenges would be the greater standardisation of RRT activation by routinely using standardized checklists to assist in structuring emergency care management.

While members of the nursing team are usually caring for patients for the duration of their shift most other staff involved may have just transiently entered the ward, and may not have the required competencies. It is incumbent on the first responder to achieve initial stabilisation, best accomplished by using the established airway, breathing and circulation management protocols (i.e. ABCDE).. Therefore, checklists requiring advanced diagnostic and therapeutic skills cannot be activated when a crisis is recognized. More advanced diagnostic and therapeutic interventions can only be provided when more members of the impromptu team arrive. The team’s leadership may then need to be re-defined and a reassessment performed using a secondary checklist.

Simulation for testing and training for RRTs and Cardiac Arrest Teams is well established [32–35]. We found testing of checklists in high-fidelity simulation highlighted important differences between patient crises experienced on general wards as compared to templates used elsewhere (i.e. in operating theatres or non-medical settings) due to variable expertise, resources and limited organiztaional support.

Checklists for emergency management have been used for years by individual clinicians as personal aides de memoire, and health care administrators have encouraged the adoption of checklists in the hope that they will minimize the risk, increase patient safety and cost of litigation [36]. However, as experience with the WHO surgical checklist has demonstrated, the benefits of checklists are only realised when the clinical staff are engaged and they are used to change the dynamics of a team’s culture [37]. Medical checklists are more likely to follow a predictable course if they make clinical sense to providers, have clearly defined endpoints [38] and actively engage the teams using them [39].

Checklists should thus not be regarded as ‘magic bullets’. However they can help minimize variation and standardize care, maintain consistency and ensure quality of care resulting in reduced complication rates and lower mortality [12, 13]. Many clinicians, however, worry that checklists may limit their clinical judgment, autonomy, and disrupt professional relationships [40]. These concerns will require significant changes in organisational culture and take time to appreciate and overcome [41–43]. Additionally investment in training will be required to embed the new checklist tools into clinical operations [44]. A vital factor in their successful use is the creation of egalitarian and flattened hierarchical team structures, so that junior team members have ‘permission to challenge’ and feel psychological safety when raising challenging issues about improving the care processes [45].

## Conclusions

The successful implementation of crisis emergency checklists has the potential to improve patient care and outcomes. This study reports on the development of consensus recommendations to provide clinicians with rapidly accessible, standardized emergency crisis checklists to assist in structuring emergency management of patient on the general medical patient wards.

Hospitals are faced with the challenge of improving reliability of their care and patient outcomes especially when treating unstable patients. The concept of emergency crisis checklists is an attractive new addition to the expanding toolkit for continuous quality improvement by clinical teams. RRS crisis emergency checklists are likely to be effective when they are performed as a team routine in the context and readiness for change. An organizational culture that values improving outcomes is essential for sustained uptake and sustained implementation of checklists. The success of checklists will depend on uptake and acceptance by providers, supported by a strongly motivated and committed team ethos. We have drawn on results from a large international learning collaborative team from the US and Europe, comprised of medical and non-medical experts and including specialists from aviation and information technology. Future research required includes systematic evaluation of these recommendations.

## Abbreviations

CRD: Centre for Reviews and Dissemination
EMBASE: Excerpta Medica dataBASE
ICU: Intensive Care Unit
iSRRS: International Society for Rapid Response Systems
MEDLINE: Medical Literature Analysis and Retrieval System Online
NEWS: National Early Warning Score
OR: Operating Room
RRS: Rapid Response System
RRT: Rapid Response Team
UK: United Kingdom
WHO: World Health Organisation
